# The Impact of Varying Enzymatic Pretreatment Durations of Wheat Gluten on the Flavour Characteristics of High-Moisture Plant-Based Extrudates

**DOI:** 10.3390/foods15050912

**Published:** 2026-03-06

**Authors:** Xiaodong Li, Huihui Dai, Boning Mao, Hongzhou An, Yanhong Bai, Lovedeep Kaur

**Affiliations:** 1 College of Food and Bioengineering, Zhengzhou University of Light Industry, Zhengzhou 450001, China; xdli0610@163.com (X.L.); b.mao@zzuli.edu.cn (B.M.); 2 College of Food Science and Technology, Henan University of Technology, Zhengzhou 450001, China; daihuihui920@haut.edu.cn; 3 School of Food and Advanced Technology, Massey University, Palmerston North 4442, New Zealand; l.kaur@massey.ac.nz

**Keywords:** wheat gluten, enzymatic pretreatment, high-moisture plant-based extrudates, flavour characteristics, sensory evaluation, taste, odour

## Abstract

This study examined the effects of varying enzymatic pretreatment durations (0–80 min) of wheat gluten on flavour characteristics of high-moisture plant-based extrudates (HMPEs). Through a comprehensive analysis involving sensory evaluation, electronic tongue, free amino acid (FAA) profiling, electronic nose, and headspace solid-phase microextraction-gas chromatography-mass spectrometer (HS-SPME-GC-MS) analysis of volatile odour compounds, it was found that HMPEs with moderate enzymatic pretreatment (40 min) achieved the highest overall sensory score. Electronic tongue and FAA results confirmed a significant enhancement in umami and sweetness, while electronic nose effectively discriminated differences in odour profiles. Extending pretreatment durations gradually reduced beany off-flavours substances (hexanal reduced by up to 174.7 μg/kg) and encouraged the formation of meaty aroma compounds (furans and pyrazines). However, excessive pretreatment (>40 min) reduced acceptance due to burnt odour caused by the excessive accumulation of pyrazines, particularly 2,3-diethyl-5-methylpyrazine. Six key volatile odour compounds were identified by integrating the analysis of variable importance projection (VIP ≥ 1) and relative odour activity value (ROAV ≥ 1), offering a foundation for targeted flavour regulation in HMPEs.

## 1. Introduction

With the global population steadily increasing and consumers becoming more focused on health, environmental protection, and sustainable diets, plant protein-based meat analogues are rapidly expanding in the market as a viable alternative to traditional animal meat [1]. Compared to conventional meat, plant protein-based meat analogues offer several benefits, such as reduced cholesterol intake, lower carbon emissions, and enhanced resource efficiency [2]. High-moisture extrusion technology is the leading technique for creating meat-like fibrous structures from plant proteins, producing products with textures and tastes similar to animal muscle, while also offering improved safety and nutritional benefits [3]. However, as consumer expectations evolve, modern consumers are concerned not only with nutritional value but also with the overall sensory experience, particularly the flavour profile, which has become a crucial factor influencing market acceptance [4,5].

The flavour quality of plant-based meat analogues remains a notable challenge. Those meat analogues, often produced by soybean or pea proteins due to their high yield and rich essential amino acids, are usually associated with undesirable off-flavours, particularly a pronounced beany off-flavour, and their overall flavour profile differs markedly from authentic meat [6,7]. Current research primarily aims to eliminate these off-flavours and develop desirable meat-like flavours. In terms of eliminating off-flavours from raw materials, heat treatment, including conventional heating, microwave [8], radio frequency [9], and other techniques, can directly remove off-flavours at the source or indirectly reduce the formation of off-flavours precursors by inhibiting lipoxygenase activity. Additionally, fermentation [10], enzymatic hydrolysis [11], and physical embedding technologies [12] can convert or mask volatile off-flavours components. During the processing of extruded plant-based meat analogues, proteins, lipids, polysaccharides, and other components in the raw materials undergo complex chemical reactions, such as the Maillard reaction, lipid oxidation, Strecker degradation, caramelization, and thiamine degradation, under high-temperature and high-pressure conditions. These reactions generate numerous volatile odour components that can effectively eliminate or suppress off-flavours and impart a pleasant aroma [4]. Furthermore, adding small amounts of exogenous flavour additives, including protein hydrolysates, yeast extracts, Maillard reaction precursors (reducing sugars, amino acids, nucleotides, and thiamine), natural spices, and vegetable oils, can effectively mask off-flavours, develop a meat-like flavour, and enhance the taste of extruded plant-based meat analogs [12,13,14,15,16].

Enzymatic hydrolysis is a highly effective method for modifying plant proteins, widely applied to enhance their functional properties [17]. Recent studies indicate that controlled enzymatic hydrolysis of proteins not only releases taste-active peptides and free amino acids, which contribute directly to desirable tastes such as umami and sweetness, but also supplies abundant precursors for the Maillard reaction, facilitating the generation of meaty aroma compounds during subsequent processing [18]. However, most existing studies focused on using protein enzymatic hydrolysates to prepare meaty Maillard flavor bases via thermal reaction with reducing sugars [14,18], rather than being used as a high-proportion raw material directly for high-moisture extrusion, and its comprehensive impact on the flavour quality of extruded plant-based meat analogs remains insufficiently explored. In our previous research, wheat gluten with varying durations of enzymatic pretreatment (0–80 min, degree of hydrolysis (DH) 0–22%) was blended with soy protein isolate at a 4:6 ratio to prepare high-moisture plant-based extrudates (HMPEs). We found that only moderate enzymatic pretreatment (40 min, DH 12.36%) of wheat gluten effectively addressed the challenge of screw conveying caused by high viscosity during high-moisture extrusion, and also regulated the fibrous structure of HMPEs [19]. Unlike the conventional exogenous addition of small amounts of flavour additives [12,13,14,15,16], this endogenous modification strategy involved a high proportion of wheat gluten enzymatic hydrolysates as raw materials to prepare HMPEs, which will provide abundant peptides and free amino acids as flavor precursors. Meanwhile, this approach has been proven to reduce mechanical energy input and alter extrusion processing conditions [19]. These factors will synergistically influence flavor development together, particularly through modulation of the Maillard reaction pathways. However, the composition and content of these precursors is closely linked to the enzymatic pretreatment time, and insufficient or excessive pretreatment may fail to improve or even deteriorate the flavour. Yet, relevant flavor research has not been systematically investigated.

Therefore, this study aims to systematically elucidate the effect of varying enzymatic pretreatment durations (0 min, 20 min, 40 min, 60 min, and 80 min) of wheat gluten on the overall flavour quality of HMPEs. We will thoroughly examine how enzymatic pretreatment of wheat gluten influences the taste and odour characteristics of HMPEs through a combination of sensory evaluation, electronic tongue analysis, free amino acid analysis, electronic nose analysis, and headspace solid-phase microextraction-gas chromatography-mass spectrometer (HS-SPME-GC-MS). Additionally, key odour components responsible for changes in the odour characteristics of HMPEs will be identified using cluster analysis, partial least squares-discriminant analysis, variable importance projection (VIP) analysis, and relative odour activity value (ROAV) analysis of volatile odour components in HMPEs. This research investigates the application of enzymatic pretreatment technology on wheat gluten, aiming to provide a theoretical foundation and technical support for developing the next generation of plant-based meat analogs with both ideal texture and superior flavour.

## 2. Materials and Methods

### 2.1. Materials

Soy protein isolate and wheat gluten were sourced from Yihai Kerry (Shanghai, China). Neutrase (50,000 U/g, BR grade) and flavourzyme (30,000 U/g, BR grade) were supplied by Soleibo Technology Co., Ltd. (Beijing, China) and used directly without further purification. Cyclohexanone was obtained from McLean Biochemical Technology Co., Ltd. (Shanghai, China). Sulfonylsalicylic acid was acquired from Kemio Chemical Reagent Co., Ltd. (Tianjin, China).

### 2.2. Enzymatic Pretreatment of Wheat Gluten

The enzymatic pretreatment of wheat gluten was conducted following our previous research [20]. Briefly, wheat gluten was dissolved in water at a concentration of 35% (*w*/*v*). The pH-value of the mixture was adjusted to 7.0, and the temperature was maintained at 30 °C. The reaction commenced with the simultaneous addition of neutral protease and flavourzyme with specific activities of 250 U/g and 150 U/g to the substrate, respectively. The reaction times were set at 0, 20, 40, 60, and 80 min. Subsequently, the mixture was placed in a boiling water bath for 10 min to deactivate the enzymes. Finally, the obtained wheat gluten hydrolysates were freeze-dried and ground into powder for further use. The DH of wheat gluten hydrolysates for the respective time points were as follows: 20 min (8.91%), 40 min (12.11%), 60 min (13.39%), and 80 min (14.43%), meanwhile, the molecular weight distribution showed a corresponding shift toward lower molecular weight peptides, which have been all determined in our previous research [20].

### 2.3. Preparation of High-Moisture Plant-Based Extrudates (HMPEs)

The HMPEs were produced using a co-rotating twin-screw extruder (CLEXTRAL Ev025, Clestero Co., Ltd., Firminy, France), featuring a screw diameter of 25 mm and a length-to-diameter ratio of 24:1, in accordance with the methodology outlined in our previous study [19]. The raw material blend, consisting of soy protein isolate and wheat gluten hydrolysates at a dry basis ratio of 6:4, was processed at a screw speed of 280 rpm and a feed rate of 4.6 kg/h, with the mixture’s moisture content maintained at approximately 57%. The extruder’s barrel was divided into six zones, with the temperature profile set at 30, 90, 120, 140, 150, and 160 °C from zone I to VI, respectively. A cooling die with dimensions of 30 mm × 4 mm was affixed to the extruder outlet to reduce the product’s temperature to 60 °C.

### 2.4. Sensory Evaluation

The sensory evaluation of HMPE was conducted in the sensory evaluation labortory of the College of Food Science and Technology at Henan University of Technology, and obtained approval from its Institutional Ethics Committee (Approval No. HAUTEC-2025-32). The panel comprised 12 assessors, all postgraduates in food science who had undergone professional sensory training. The HMPEs were uniformly cut into pieces measuring 5 cm × 5 cm and were randomly assigned three-digit codes for the five samples. Assessors were provided with disposable plates, gloves, and mouthwash, and were required to rinse their mouths thoroughly between sample evaluations to minimise cross-over effects. The samples were scored according to the criteria outlined in Table S1, with attributes such as fibrous structure, texture, colour, taste, and odour being assessed.

### 2.5. Electronic Tongue Analysis

The HMPEs were ground following rapid freezing with liquid nitrogen. Subsequently, 10.00 g of the powdered HMPE was combined with 40 mL of deionized water using a vortex mixer, then shaken for 30 min at room temperature for extraction. The mixture underwent centrifugation at 10,000× *g* for 10 min, and the supernatant was collected. The extraction process was repeated by adding another 40 mL of deionised water to the residue. The combined supernatants were filtered and adjusted to a final volume of 100 mL. This prepared solution was transferred into a dedicated electronic tongue beaker for taste profiling using an electronic tongue system (MOS ATREE, Alpha MOS Co., Ltd., Toulouse, France). The measurement conditions were set as follows: 120 s for signal acquisition and 10 s for sensor cleaning, with four replicated determinations per sample [21].

### 2.6. Determination of Free Amino Acids in HMPEs

Free amino acids were extracted and determined following the method outlined by Luo et al. [22]. Precisely 0.5 g of powdered HMPE was mixed with 5 mL of distilled water. The mixture underwent vortexing and was then shaken for 1 h. After centrifugation at 6000× *g* for 20 min, 400 µL of the supernatant was combined with 100 µL of 10% sulfosalicylic acid. This mixture was left at 4 °C for 60 min to ensure complete protein precipitation. It was subsequently centrifuged at 12,000× *g* for 10 min. The supernatant obtained was centrifuged again under identical conditions to achieve clarity. Finally, the supernatant was diluted to the required concentration, filtered through a 0.45 µm aqueous phase membrane, and analysed using an automatic amino acid analyser (S-433D, Sykam GmbH, Munich, Germany).

### 2.7. Electronic Nose Analysis

The odour profiles of the HMPEs were examined using an electronic nose (Fox 4000, Alpha M.O.S., Toulouse, France). For each measurement, precisely 2.00 g of the powdered HMPE was weighed and placed into a 20 mL headspace vial. The analysis was conducted under these conditions: the headspace vial was heated to 60 °C with an equilibration time of 5 min, after which 2.5 mL of the headspace gas was injected into the system. The sensor response was recorded for 120 s. To prevent cross-contamination between samples, a system cleaning step lasting 200 s was carried out after each analysis [23].

### 2.8. Analysis of Volatile Odour Compounds in HMPEs by HS-SPME-GC-MS

Volatile odour compounds in HMPEs were extracted using HS-SPME, following a modified method from the literature [24]. Briefly, 2.00 g of powdered HMPE was placed in a 20 mL headspace vial, to which 10 µL of the internal standard (cyclohexanone) was added, and the vial was sealed. The vial was equilibrated at 60 °C before a pre-conditioned 50/30 μm DVB/CAR/PDMS fibre (conditioned at 250 °C for 30 min, SUPELCO Analytical, Inc., Bellefonte, PA, USA) was exposed to the sample for 30 min to allow adsorption. Subsequently, the fibre was inserted into the GC inlet and desorbed at 250 °C for 5 min. GC-MS (A 7890 GC equipped with a 7000D MS detector, Agilent Technologies, Inc., Santa Clara, CA, USA) analysis was conducted using an HP-5 MS capillary column (30 m × 250 μm × 0.25 μm. Agilent Technologies, Inc.) with helium as the carrier gas at a flow rate of 1.8 mL/min in splitless mode. The oven temperature program was as follows: initially held at 40 °C for 5 min, then increased to 130 °C at 2 °C/min with a 5 min hold, and finally raised to 250 °C at 10 °C/min with another 5 min hold. MS detection utilised electron impact ionisation at 70 eV, with ion source and quadrupole temperatures set at 230 °C and 150 °C, respectively, scanning *m*/*z* 33–400 in full scan mode.

Volatile odour compounds were identified through the NIST database and retention index, which was calculated from n-alkanes. Their relative contents were quantified using the internal standard method, as per the following formula:

(1) $$C=\frac{A\times C_{S}\times V_{S}}{A_{S\times m}}$$

Note: *C*, relative contents of volatile odour compounds; *C_S_*, concentration of the internal standard; *A*, peak area of the sample; *A_S_*, peak area of the internal standard; *V_S_*, volume of the internal standard; *m*, weight of the sample.

Further analysis employed cluster analysis, orthogonal partial least squares-discriminant analysis (OPLS-DA), and relative odour activity value (ROAV) on the relative contents of the odour compounds to identify and screen key differential compounds. The ROAV was calculated as follows:

(2) $$ROAV\approx 100\times \frac{C}{T}\times \frac{T_{S}}{C_{S}}$$

Note: *C* and *T*, relative contents and sensory threshold of volatile odour compounds; *C_S_* and *T_S_*, relative contents and sensory threshold of normalized reference volatile odour compounds [25].

### 2.9. Statistical Analysis

Data are presented as mean ± standard deviation. Statistical analysis was performed using SPSS statistical software (IBM, Version 20, New York, NY, USA). All quantitative data were tested for normality and homogeneity of variance. For data that did not meet the assumptions for parametric tests, the non-parametric Kruskal-Wallis H test was applied, followed by Dunn’s test for post hoc multiple comparisons (*p* < 0.05). Descriptive sensory data were analyzed using a two-way mixed-model ANOVA with samples as the fixed factor and assessors as the random factor. Multivariate statistical analysis of Electronic tongue and Electronic nose data were analyzed by their built-in software. Orthogonal OPLS-DA of colatile odour compounds was performed using the online MetaboAnalyst 6.0 platform (https://www.metaboanalyst.ca/, accessed on 15 December 2025).

## 3. Results and Discussion

### 3.1. Sensory Evaluation

A thorough sensory analysis based on subjective evaluation was conducted to assess the effects of different enzymatic pretreatment durations of wheat gluten on the quality of HMPEs. This section initially performed sensory evaluation of the fibrous structure, texture and colour of HMPEs, attempting to objectively verify the finding of our previous studies that moderate enzymatic pretreatment achieved the optimal texture structure of HMPE [19]. Subsequently, sensory analysis was carried out to evaluating the taste and odour characteristics of HMPEs, which was the core research focus of this study.

**Figure 1:**
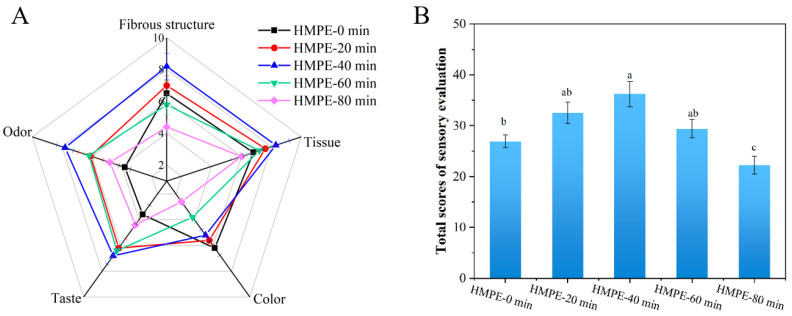
Sensory evaluation radar chart (**A**) and overall sensory scores (**B**) of the HMPEs obtained with varying enzymatic pretreatment durations of wheat gluten. Different lowercase letters in figure (**B**) mean significant differences (*p* < 0.05).

As illustrated in Figure 1A, the sensory scores for the fibrous structure of HMPEs initially increased and then decreased with the enzymatic pretreatment durations of wheat gluten extended from 0 to 80 min. This pattern was largely due to the varied effects of enzymatic hydrolysis on the protein network structure. Moderate enzymatic pretreatment of wheat gluten effectively mitigated the excessively dense fibrous structure caused by the high texturization degree in HMPEs without such pretreatment. Conversely, excessive enzymatic pretreatment could severely impair wheat gluten’s ability to form a three-dimensional network, significantly diminishing the viscoelasticity of the protein melt during extrusion. This led to a very low degree of texturization and an overly loose fibrous structure, ultimately obstructing the formation of an ordered fibrous structure [19]. Similarly, the texture scores of HMPEs also showed an initial rise followed by a decline with increasing enzymatic pretreatment time, peaking at 40 min. The HMPE without enzymatic pretreatment exhibited excessive hardness and poor elasticity, which was mainly caused by the overly dense and rigid fibrous structure resulting from the highest texturization degree. In contrast, moderate enzymatic pretreatment (e.g., 40 min) achieved balanced texturization, effectively disrupting excessive protein cross-linking and enhancing hardness and elasticity with a desirable fibrous structure. However, excessive enzymatic pretreatment severely degraded wheat gluten proteins, considerably weakening the protein network integrity and significantly reducing the viscoelasticity of the protein melt during extrusion. Consequently, the material failed to form well-organised fibrous, resulting in insufficient texturization, an overly soft texture, and notably reduced elasticity [19,26]. The results of fiber structure and texture objectively confirmed that moderate enzymatic pretreatment achieved the optimal texture structure of HMPE. The colour sensory score of HMPEs declined progressively with longer enzymatic pretreatment of wheat gluten. This change resulted mainly from the enzymatic hydrolysis of wheat gluten, producing abundant free amino acids that underwent Maillard reactions during extrusion, darkening the product. This interpretation was supported by our earlier observations that L* values decreased while a* and b* values increased of HMPEs [19].

Regarding the sensory evaluation of flavour characteristics of HMPEs, both the sensory scores for odour and taste initially increased and then decreased as the enzymatic pretreatment duration for wheat gluten extended, peaking at 40 min. This pattern might be attributed to the production of small peptides and free amino acids during the enzymatic pretreatment of wheat gluten. These compounds not only directly enhanced desirable taste components, such as umami and sweetness, but also participated in Maillard reactions during extrusion, producing roasted aroma substances that effectively masked beany off-flavours and improved odour acceptability [18]. However, excessive enzymatic pretreatment led to an over-accumulation of amino acids and peptides, which in turn promoted overly intense Maillard reactions. This resulted in burnt or bitter substances that adversely affected both the odour and taste characteristics of HMPEs [4].

In Figure 1B, the overall sensory evaluation score revealed that the HMPE obtained with 40 min of wheat gluten enzymatic pretreatment achieved the highest score. This indicated that moderate enzymatic pretreatment of wheat gluten could significantly enhance the texture and flavour of HMPE. This was essentially attributed to the moderate DH (12.11%) of wheat gluten and the shifted molecular weight distribution toward low-molecular-weight peptides [20], which avoided the drawbacks of insufficient hydrolysis (0–8.91% DH) and excessive hydrolysis (13.39–14.43% DH), while optimizing the protein network for desirable texture and providing abundant taste-active peptides and flavor precursors for improved taste and odour. However, given the subjective nature of sensory evaluations and the fact that changes in flavour characteristics are inherently linked to alterations in taste and odour substances, further investigation is warranted. The underlying material basis for changes in the taste and odour properties of HMPEs resulting from varying durations of wheat gluten enzymatic pretreatment will be thoroughly explored using electronic tongue analysis, free amino acid content determination, electronic nose analysis, and quantification of volatile odour compounds. Notably, this descriptive sensory analysis result only revealed the impact of various enzymatic pretreatment durations of wheat gluten on the objective sensory characteristics of the HMEPs. Future research should recruit a larger consumer group for preference testing based on this foundation, in order to directly evaluate the market acceptance potential of products and establish a correlation model between descriptive attributes and consumer preference.

### 3.2. Electronic Tongue Analysis

**Figure 2:**
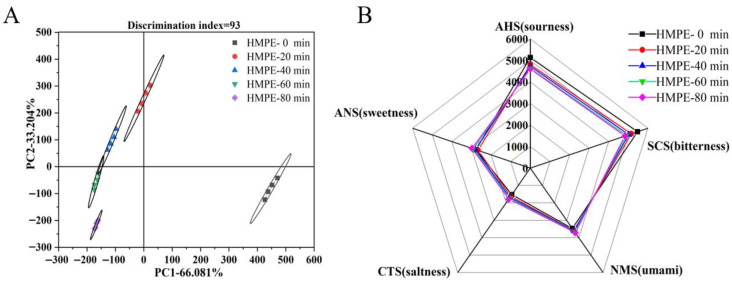
PCA (**A**) and radar fingerprint (**B**) of electronic tongue data for HMPEs obtained with varying enzymatic pretreatment durations of wheat gluten.

An electronic tongue was utilised to objectively assess the impact of varying enzymatic pretreatment durations of wheat gluten on the taste properties of HMPEs. The electronic tongue mimics human taste perception by generating specific responses to taste compounds through its five fundamental taste sensors: sourness, sweetness, bitterness, saltiness, and umami [27]. The collected sensor response values of HMPEs were analyzed using principal components analysis (PCA) to systematically evaluate the differences in taste properties among HMPEs obtained with varying enzymatic pretreatment duration of wheat gluten. Subsequently, a radar chart was then plotted to depict the taste profiles of the HMPEs based on the response values, with the results displayed in Figure 2.

The PCA results depicted in Figure 2A indicated that the first two principal components of PC1 and PC2 explained 66.081% and 33.204% of the total variance, respectively, with a cumulative contribution rate of 99.285% (>85%). This suggested that these components collectively capture the majority of the core taste-related information of HMPEs subjected to varying durations of wheat gluten enzymatic pretreatment [22]. In the PCA plot, the taste response distribution areas for the four groups of HMPEs, each subjected to different enzymatic pretreatment times, were markedly distant from the non-enzymatic pretreatment group, indicating a significant difference in taste properties of HMPEs with or without wheat gluten enzymatic pretreatment. Additionally, among the four enzymatic pretreatment (20 min, 40 min, 60 min and 80 min) groups, the taste response distribution areas of HMPEs were arranged systematically along the negative axes of PC1 and PC2, with relatively small distances between. This phenomenon indicated that varying the duration of enzymatic pretreatment of wheat gluten significantly affected the taste properties of HMPEs, albeit less so than the presence or absence of enzymatic pretreatment. Meanwhile, only a partial overlap was observed in the taste response distribution areas between the HMPEs obtained with the time of enzymatic pretreatment at 40 min and 60 min, indicating a certain similarity in taste properties.

Radar chart analysis (Figure 2B) further revealed that while the five groups of HMPEs exhibited generally similar taste profiles, distinct differences emerged in sensor response values across the samples. Notably, as the enzymatic pretreatment time of wheat gluten increased, the sensor response values of umami, saltness, and sweetness of HMPEs gradually increased, whereas the sourness values decreased consistently, and bitterness values initially fell before rising again. The enhanced umami and sweetness response values improved the taste characteristics of HMPEs, accounting for the higher taste scores observed in sensory evaluations. This improvement might be attributed to the abundant peptides and amino acids generated by enzymatic hydrolysis of wheat gluten using complex enzymes (neutral protease and flavourzyme). These enzymes not only boosted the levels of positive taste components, such as sweet and umami amino acids, but also effectively reduced the formation of negative taste components, such as bitter peptides [28]. However, excessive enzymatic hydrolysis of wheat gluten (beyond 40 min) might inadvertently increase the concentration of bitter compounds. Additionally, the large amounts of small peptides and free amino acids can undergo Maillard reactions under the high-temperature and high-pressure conditions of subsequent high-moisture extrusion, leading to the formation of new bitter substances [4]. This results in a resurgence of bitter response values and taste sensory scores. The specific alterations in these taste-active compounds, particularly free amino acids, are examined in the following section.

### 3.3. Free Amino Acids Analysis

Free amino acids (FAAs) play a significant role in shaping the overall flavour profile of food, not only through their inherent taste properties and synergistic interactions with other flavour compounds, but also by serving as essential flavour precursors that undergo transformations during food processing [22]. Building on the previous sensory evaluation and electronic tongue analysis of HMPEs, this study further investigated the composition and content of FAAs in HMPEs, aiming to elucidate the molecular basis for taste changes in HMPEs subjected to varying durations of wheat gluten enzymatic pretreatment. The results are summarized in Table 1.

The FAAs in HMPEs produced without enzymatic pretreatment of wheat gluten predominantly comprised Lys, Arg, Asn, and Asp, in that order. However, following enzymatic pretreatment, the main amino acids were Lys, Ile, Asn, and Tyr. This shift might be primarily attributed to the specific cleavage of the compound proteases (neutral protease and flavourzyme), which preferentially hydrolyzed peptide bonds containing hydrophobic and aromatic amino acids [29]. Meanwhile, as the enzymatic pretreatment durations increased from 0 to 80 min, the total free amino acid contents in the HMPEs increased significantly from 409.27 mg/100 g to 844.69 mg/100 g. This marked increase indicated effective protein degradation and the release of FAAs induced by enzymatic pretreatment, enriching the taste components of HMPEs, which aligned with the enhanced taste intensity detected by the electronic tongue. From the perspective of taste attributes, both umami and sweetness amino acids showed a continuous increase with prolonged enzymatic pretreatment time, directly supporting the rising umami and sweetness sensor response values observed in the electronic tongue. Notably, although the total content of bitter amino acids also exhibited an upward trend, sensory evaluation and electronic tongue analysis revealed a decrease followed by an increase in bitter taste perception. This phenomenon could be attributed to the effective control of bitter peptide generation by complex enzymes during early hydrolysis, the masking effect of abundant umami and sweet amino acids, and the fact that bitter perception was a complex integration of multiple taste signals [18]. However, with excessive enzymatic pretreatment of wheat gluten, the accumulated bitter amino acids and peptides, as well as new bitter substances formed during the process of extrusion, might eventually exceed the masking threshold, leading to increased bitterness perception [4]. It should be noted that, in addition to free amino acids, taste peptides generated during enzymatic hydrolysis and their significant synergistic interaction with free amino acids also played a crucial role in influencing the overall taste profile [30]. However, due to the complexity of peptide composition in the enzymatic hydrolysates and the further intricate alterations these peptides undergo under the high-temperature and high-pressure conditions of extrusion processing, a detailed analysis of their specific composition and taste contribution was beyond the scope of this study.

### 3.4. Electronic Nose Analysis

**Figure 3:**
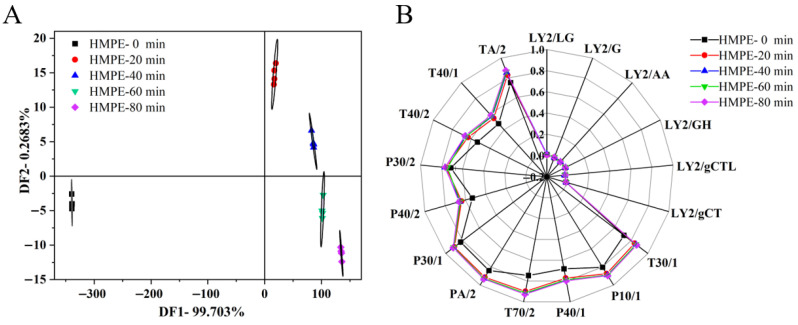
DFA (**A**) and radar fingerprint (**B**) of electronic nose data for HMPEs obtained with varying enzymatic pretreatment durations of wheat gluten.

In this study, an electronic nose equipped with 17 specific odour sensors was utilised to objectively assess the impact of varying enzymatic treatment durations on the odour properties of HMPEs. The electronic nose mimics human olfactory perception by generating specific responses to odour compounds through its sensors, thereby reflecting the overall odour characteristics of samples and avoiding the biases inherent in human evaluation [24]. Sensor response values for odour components in HMPEs were collected and analysed using discriminant factor analysis (DFA) to systematically evaluate the differences in odour properties among HMPEs derived from wheat gluten enzymatic pretreatment at different stages. A radar chart was then plotted to illustrate the odour profiles of the HMPEs based on these response values, with the results presented in Figure 3.

DFA results in Figure 3A revealed that the differentiation indexes of DF1 and DF2 were 99.703% and 0.268%, respectively, and the cumulative contribution rate of DF1 and DF2 reached 99.971% (>85%), which indicated that these two factors adequately captured the core odour characteristics of HMPEs subjected to varying durations of wheat gluten enzymatic pretreatment [31]. In the DFA plot, the odour response distribution areas for the five groups of HMPEs were distinctly separated, with no overlap, highlighting significant differences in odour properties based on the pretreatment duration. Notably, a clear distinction was evident between the four enzymatic pretreatment groups and the non-enzymatic group, confirming that enzymatic pretreatment of wheat gluten significantly modified HMPEs. Furthermore, among the four enzymatic pretreatment groups (20 min, 40 min, 60 min, and 80 min), the odour response distribution areas were arranged systematically along the positive DF1 and negative DF2 axes. Although these areas did not overlap, they were relatively close to each other, suggesting that the duration of enzymatic pretreatment also affected the odour properties of HMPE. However, the presence or absence of enzymatic pretreatment remained the primary factor, aligning with taste variation results from electronic tongue analysis.

As shown in the radar chart (Figure 3B), the six L-series odour sensors exhibited no significant response to the odour substances in HMPEs, whereas the P- and T-series sensors showed marked responses. Notably, the odour profiles of HMPEs varied significantly with different durations of wheat gluten enzymatic pretreatment. The odour profile ranges for the four enzymatic pretreatment groups extended beyond that of the non-enzymatic pretreatment group, indicating that enzymatic pretreatment of wheat gluten enhanced the overall odour intensity of HMPEs. However, as the enzymatic pretreatment duration increased from 20 to 80 min, the expansion of the odour profile range was limited, suggesting that the differences in odour intensity due to varying pretreatment times were less pronounced than those between enzymatic and non-enzymatic pretreatment. This observation aligned with sensory evaluation results. Differences in odour profiles between enzymatically and non-enzymatically pretreated HMPEs were evident in the response values of all odour sensors except the L-series. In contrast, variations among the four enzymatic pretreatment groups were primarily reflected in the response values of sensors such as TA/2, T40/1, T40/2, P40/2, T70/2, PA/2, and T30/1, indicating significant changes in the content of organic, polar, and aromatic compounds. These changes were likely due to small peptides and free amino acids produced during the enzymatic pretreatment of wheat gluten, which underwent Maillard reactions under high-temperature and high-pressure conditions during extrusion, generating more odour substances. It is important to note that while the electronic nose effectively identified changes in the odour profile, it could not precisely quantify specific odour compounds or their correlation with odour property changes. Therefore, GC-MS should be utilised in future analyses to elucidate the detailed composition of volatile odour compounds and uncover the reasons for changes in odour properties of HMPEs due to varying enzymatic pretreatment durations of wheat gluten.

### 3.5. Qualitative and Quantitative Analysis of Volatile Odour Compounds

A qualitative and quantitative analysis of volatile odour compounds in HMPEs, derived from wheat gluten with varying enzymatic pretreatment durations, was performed using SPME-GC-MS. A total of 32 volatile odour compounds were identified and quantified in different HMPEs, including 6 aldehydes, 3 ketones, 4 alcohols, 7 furans, 3 thiophenes, and 9 pyrazines, as summarized in Table 2.

Aldehydes, as key aroma compounds in foods, are primarily derived from lipid oxidation and the Maillard reaction. Given their typically low odour thresholds, even minimal aldehyde concentrations can significantly impact a product’s overall odour profile [32]. The results in Table 2 demonstrated significant changes in the relative content of aldehydes in HMPEs with prolonged enzymatic pretreatment durations of wheat gluten. Notably, the content of fatty aldehydes, represented by the grassy-smelling hexanal, exhibited a consistent downward trend. Hexanal, the main contributor to the beany off-flavour [6], showed the most substantial reduction, with a maximum decrease of 174.7 μg/kg, effectively diminishing the beany off-flavours in HMPEs. Conversely, the levels of aromatic aldehydes like benzaldehyde and phenylacetaldehyde, primarily formed through the oxidative degradation of proteins or amino acids [33], increased significantly. These compounds impart roasted almond and rose aromas, respectively, enhancing the odour properties of HMPEs. This compositional shift can be attributed to several factors. Firstly, the extended enzymatic pretreatment of wheat gluten gradually meant a gradual increase in DH (0–14.43%) and the aggregation of molecular weight distribution towards low molecular weight peptides, which reduced the viscosity of material during extrusion, decreasing mechanical energy input and mitigating thermal effects, thereby suppressing lipid oxidation [19,20]. Secondly, polypeptide compounds released during the enzymatic pretreatment exhibited certain antioxidant activity, further inhibiting lipid oxidation [34]. Additionally, the increased availability of aromatic free amino acids, such as phenylalanine in the hydrolysates, provides abundant precursors for Strecker degradation, promoting the formation of aromatic aldehydes.

Ketone compounds, such as the typical lipid oxidation products 2-nonanone and 2-decanone, were often reported to impart buttery and cheesy notes [35], potentially enhancing the meat-like aroma of products. However, the content of these ketone compounds decreased with extended enzymatic pretreatment durations of wheat gluten, and their high sensory thresholds limited their overall contribution to odour properties. In terms of alcohol compounds, the content of saturated alcohols like 3-methyl-butanol increased significantly, rising by up to approximately 16 times. Despite this increase, the influence of 3-methylbutanol on odour properties was restricted by its generally high odour thresholds. Notably, the content of unsaturated alcohols, particularly 1-octen-3-ol, which was known for its mushroom aroma and significant role in beany off-flavours [6], decreased gradually with prolonged enzymatic pretreatment of wheat gluten. Additionally, the odour threshold of 1-octen-3-ol was relatively low (1.5 μg/kg), which would have a positive significance in weakening the beany off-flavours of the HMPEs.

The analysis of furan compounds indicated that 2-pentylfuran, with its buttery and meaty aroma, predominated among the seven furan compounds in HMPEs, followed by maltol, which has a caramel-like odour. Notably, both 2-pentylfuran and maltol possess relatively low thresholds, significantly enhancing the odour characteristics of HMPEs [36]. Furthermore, the relative content of all furan compounds increased to varying extents as the enzymatic pretreatment duration for wheat gluten was extended, thereby improving the odour characteristics of HMPEs. Furan compounds typically arise from glycolysis, Maillard reactions, and the cyclization of unsaturated aldehydes, with branched furan compounds primarily forming through the Maillard reaction pathway [35]. Prolonging the enzymatic pretreatment of wheat gluten enriched the flavour precursors, such as peptides and free amino acids, available for the Maillard reaction during extrusion, thus fostering the formation of furan compounds and significantly enhancing the grilled meat aroma of the HMPEs.

Thiophene compounds are known for their characteristic sulphurous and meaty aroma, primarily resulting from the oxidative degradation of sulphur-containing amino acids, the Maillard reaction, and interactions with lipid oxidation products [37]. As shown in Table 2, the relative content of three thiophene compounds in HMPEs increased progressively with the extending enzymatic pretreatment durations of wheat gluten, enhancing the odour characteristics of HMPEs. This increase was mainly due to the rise in sulphur-containing free amino acids (such as methionine) in the enzymatic products of wheat gluten, which served as rich precursors for the formation of these thiophene compounds.

Pyrazine compounds primarily arise from the condensation of α-amino ketones, which are produced through the oxidative degradation of amino acids (especially lysine with 2 amino groups) during the Maillard reaction. These compounds are crucial for imparting nutty, grilled, or roasted flavours to food [35]. As shown in Table 2, the relative content of nine pyrazine compounds in HMPEs increased to varying extents with the extension of the enzymatic pretreatment durations of wheat gluten. Notably, the relative content of pyrazine, 2,5-dimethylpyrazine, 3-ethyl-2,5-dimethylpyrazine, and 2,3-diethyl-5-methylpyrazine, which had relatively low sensory thresholds, increased significantly. This increase was largely due to the intensified Maillard reaction, driven by the abundant free amino acids—especially lysine, the most abundant FAA (Table 1) and a highly efficient precursor for pyrazine formation—released during the enzymatic hydrolysis of wheat gluten [35]. Particularly significant is 2,3-diethyl-5-methylpyrazine, with an exceptionally low sensory threshold of 0.0031 μg/kg, enabling it to produce a strong olfactory stimulus even at minimal concentrations, thereby playing a vital role in developing roasted flavours in foods [35]. However, it is important to note that excessively high levels of low-threshold pyrazine compounds can lead to an unpleasant burnt odour. This is likely the primary reason for the decline in sensory scores for odour when the enzymatic pretreatment duration of wheat gluten exceeds 40 min, highlighting more nuanced differences in the odour properties of HMPEs.

In summary, the extent of enzymatic pretreatment of wheat gluten markedly altered the composition and relative content of volatile odour compounds, thereby significantly influencing the odour characteristics of HMPEs. A moderate pretreatment duration of 40 min could enhance the presence of compounds imparting roasted and meaty aromas, such as aromatic aldehydes, furans, and pyrazines, while effectively reducing those responsible for beany off-flavours, like hexanal and 1-octen-3-ol. Conversely, excessive enzymatic pretreatment might lead to an increase in compounds associated with a burnt odour, particularly excessive pyrazines. This shift originated from the enzymatic degradation of proteins, which systematically altered the composition and concentration of the flavour precursors available for Maillard and Strecker reactions. Notably, although the contents of most precursors (such as free amino acids) increased continuously from 0 to 80 min, providing a richer substrate for flavour-forming reactions, sensory scores declined beyond 40 min. This indicated that the final flavour quality was not linearly correlated with precursor abundance and odorant content but was governed by a balance of reaction pathways and intensities. Furthermore, our previous study showed that extended hydrolysis of wheat gluten reduced torque, pressure, and specific mechanical energy during extrusion, which would generally suppress the Maillard reaction [19]. However, the continued increase in Maillard products (e.g., furans and pyrazines) observed here suggested that the substantial rise in flavour precursors (peptides and amino acids) at higher hydrolysis degrees might exert a dominant positive influence on reaction progression.

### 3.6. Cluster Heat Map Analysis of Volatile Odour Compounds

**Figure 4:**
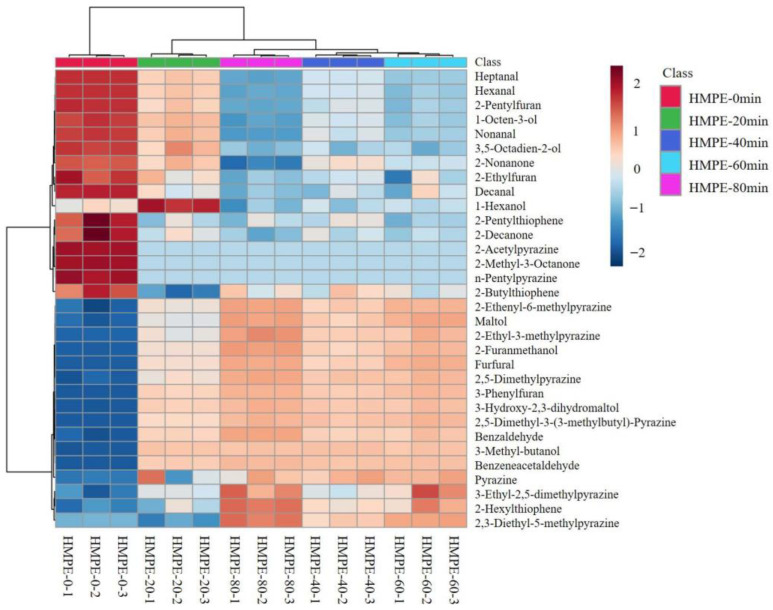
Cluster heat map analysis of volatile odour compounds in HMPEs obtained with varying enzymatic pretreatment durations of wheat gluten.

The cluster analysis heatmap of volatile odour compounds can visualize the subtle differences in the odour characteristics of HMPEs obtained with varying enzymatic pretreatment durations of wheat gluten [38]. From Figure 4, all samples are distinctly separated into two primary clusters: one comprising HMPEs without enzymatic pretreatment and the other including those with pretreatment times ranging from 20 to 80 min. This clearly demonstrated that enzymatic pretreatment was the primary factor influencing the odour characteristics of HMPEs, aligning with the odour profile changes identified by the electronic nose. Notably, sub-clustering within the enzymatic pretreatment groups indicated that the duration of pretreatment was a secondary yet significant factor. Specifically, the odour characteristics of HMPEs with 20-min and 40-min pretreatments were more similar to each other, while the 60-min and 80-min groups formed another adjacent sub-cluster. This pattern suggested a distinct phase shift in the volatile odour compounds of HWPEs when the pretreatment time exceeded 40 min, marked by a significant accumulation of Maillard reaction products, particularly furan and pyrazine compounds.

Primary and secondary clusters analysis through the cluster heat map, combined with the significant accumulation of Maillard reaction products (particularly pyrazines) in the 60 min and 80 min enzymatic groups, provides chemical substantiation for the highest odour score for the HMPE with 40 min enzymatic pretreatment of wheat gluten in sensory evaluation. While these Maillard reaction products at appropriate levels contributed desirable roasted and nutty notes for HMPEs, their excessive accumulation resulting from an over-intense Maillard reaction with rich free amino acids as precursor substances might lead to an overly intense aroma profile and even undesirable burnt off-flavours, thereby reducing overall odour acceptability.

### 3.7. OPLS-DA Analysis of Volatile Odour Compounds

**Figure 5:**
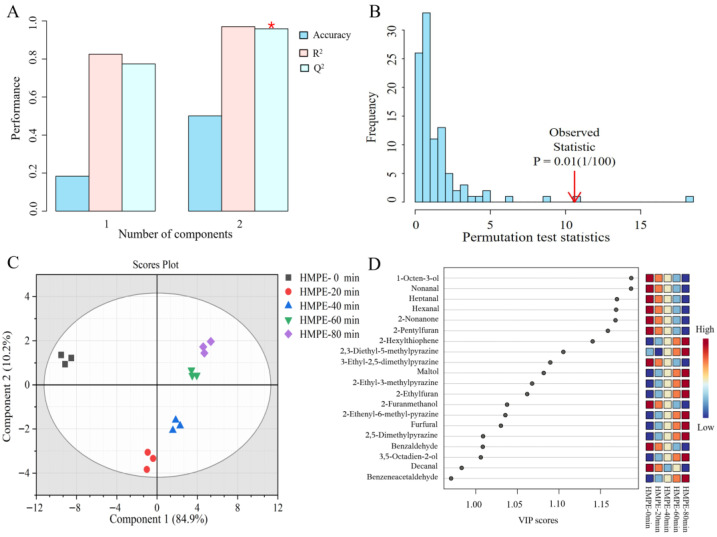
OPLS-DA analysis of volatile odour compounds in HMPEs obtained with varying enzymatic pretreatment durations of wheat gluten. (**A**) The goodness of fit (R^2^) and prediction (Q^2^) of the OPLS-DA model, the asterisk (*) indicates the selected model with two components, which exhibited optimal performance; (**B**) The significant permutation test of the OPLS-DA model; (**C**) 2D scores plot with two components in the model; (**D**) Variable importance projection (VIP) value of volatile odour compounds in HMPEs.

The OPLS-DA, effective for distinguishing sample groups and constructing discriminant models [25], was used to analyse the relative abundances of 32 volatile odour compounds in HMPEs (5 pretreatment durations, *n* = 3 per group, 15 samples in total) and to identify key odourants responsible for odour differences. The 5-fold cross-validation results of the 2-component OPLS-DA model (Figure 5A) exhibited excellent performance, with the goodness-of-fit (R^2^) and prediction ability (Q^2^) values of 0.9698 and 0.9618, respectively. Furthermore, a permutation test with 100 permutations yielded *p* < 0.01 (Figure 5B), confirming model reliability and excluding overfitting [39]. These statistical metrics collectively indicated strong explanatory power and robust predictive accuracy for identifying discriminatory compounds. The 2D score plot for the two-component model (Figure 5C) showed the HMPE without enzymatic pretreatment of wheat gluten isolated in the second quadrant, while HMPEs with 20 min enzymatic pretreatment occupied the lower part of the third quadrant and those with 40, 60, and 80 min pretreatment lay in the first quadrant. Moreover, the 40, 60, and 80 min samples clustered closely. Thus, the model fully separated the volatile profiles of the HMPEs, which was consistent with the distribution of HMPEs in the electronic nose results.

The variable importance in projection (VIP) value was used to assess each variable’s contribution in the OPLS-DA model. Variables with VIP ≥ 1 are generally regarded as significant contributors to the model [40]. As shown in Figure 5D, 18 volatile odour compounds had VIP values ≥ 1: 1-octen-3-ol, nonanal, heptanal, hexanal, 2-nonanone, 2-pentylfuran, 2-hexylthiophene, 2,3-diethyl-5-methylpyrazine, maltol, 3-ethyl-2,5-dimethylpyrazine, 2-ethyl-3-methylpyrazine, 2-ethylfuran, 2-furanmethanol, 2-ethyl-6-methylpyrazine, furfural, 2,5-dimethylpyrazine, benzaldehyde, and 3,5-octadien-2-ol. These compounds were identified as the statistically significant differential volatile compounds driving the odour profile variations of HMPEs. However, VIP analysis alone cannot directly indicate each compound’s actual odor contribution, as it overlooks sensory thresholds essential for odor perception—only exceeding the threshold can be perceived.

### 3.8. Relative Odour Activity Value (ROAV) Analysis of Volatile Odour Compounds

The contribution of volatile odour compounds to HMPEs depends not only on their contents but also on their detection thresholds, the ratio of content to threshold is therefore the key index for assessing their odour impact [41]. Therefore, the ROAV method was applied to evaluate the actual influence of each compound on the odour properties of HMPEs. In this study, 2,3-diethyl-5-methylpyrazine served as the normalised reference for ROAV calculations. Compounds with ROAV ≥ 1 were deemed essential volatile odour compounds, while those with 0.1 ≤ ROAV ≤ 1 were classified as modified volatile odour compounds [42]. The results are given in Table 3. Six essential volatile odour compounds (ROAV ≥ 1) were identified in HMPEs: hexanal, nonanal, 2-pentylfuran, maltol, 2,5-dimethylpyrazine and 2,3-diethyl-5-methylpyrazine, all of which had VIP ≥ 1. Five modified volatile odour compounds were also identified: heptanal, 2-methyl-3-octanone, 1-octen-3-ol, pyrazine and 3-ethyl-2,5-dimethylpyrazine. Among these, 2-pentylfuran (ham flavour), maltol (caramel flavour), 2,5-dimethylpyrazine (popcorn flavour) and 2,3-diethyl-5-methylpyrazine (roasted flavour) are likely the principal contributors to an ideal meat-like odour profile [35]. By contrast, hexanal (grass flavour), nonanal (citrus flavour) and 1-octen-3-ol (mushroom flavour), as well as excessive 2,3-diethyl-5-methylpyrazine (roasted flavour), are likely associated with undesirable beany off-flavours and burnt notes, respectively [6,35].

### 3.9. Screening of Key Volatile Flavour Compounds Based on VIP and ROAV

By integrating the previous analyses for VIP ≥ 1 and ROAV ≥ 1 [40], this study ultimately identified six key volatile odour compounds that exhibited both statistically significant changes and substantive contributions to the overall odour of HMPEs obtained with varying enzymatic pretreatment durations of wheat gluten: hexanal, nonanal, 2-pentylfuran, maltol, 2,5-dimethylpyrazine, and 2,3-diethyl-5-methylpyrazine. These compounds can be categorized into two functional groups: (1) Positive and synergistic contributors (2-pentylfuran, maltol, 2,5-dimethylpyrazine, and 2,3-diethyl-5-methylpyrazine) that provide desirable meaty, caramel, and roasted notes; (2) Negative contributors (hexanal, nonanal, and excess 2,3-diethyl-5-methylpyrazine) that cause beany off-flavour and burn odours. The shifting balance among these six compounds accounts for the decrease in beany off-flavour, the initial enhancement and followed by deterioration of the meat-like aroma with increasing enzymatic pretreatment. Notably, 2,3-diethyl-5-methylpyrazine, which has a roasted odour, shows an exceptionally high ROAV owing to its extremely low threshold and is therefore a likely driver of the burnt odour observed after excessive enzymatic hydrolysis.

### 3.10. Correlation Analysis Between Key Volatile Compounds and Odour Sensory Scores

Pearson correlation analysis was performed on the mean contents of the six key volatile compounds and odour sensory scores. The results showed negative correlations for hexanal (r = −0.47) and nonanal (r = −0.21), and positive correlations for 2,5-dimethylpyrazine (r = 0.47), maltol (r = 0.42), 2-pentylfuran (r = 0.38) and 2,3-diethyl-5-methylpyrazine (r = 0.24). Among negative contributors, hexanal with the highest absolute r-value was the core beany off-flavour compound, whose reduction improved odour acceptability; nonanal was a secondary off-flavour substance with weak negative correlation. For positive contributors, 2,5-dimethylpyrazine and maltol were the main sources of meaty and caramel aromas with higher positive r-values; 2,3-diethyl-5-methylpyrazine had the lowest r-value, which might be due to its excessive accumulation after 40 min caused burnt odour and offset its roasted aroma contribution.

## 4. Conclusions

This study systematically demonstrated that the enzymatic pretreatment duration of wheat gluten serves as thecritical determinant for the overall quality of HMPEs. Enzymatic pretreatment of wheat gluten for 40 min achieved an optimal balance between texture and flavour characteristics of HMPEs. Moderate enzymatic pretreatment (≤40 min) of wheat gluten enhanced umami and sweetness perception of HMPEs (through the release of more free amino acids), promoted meaty aromas (via increased furans and pyrazines) and suppressed beany off-flavour (e.g., the decreased hexanal and nonanal). However, excessive enzymatic pretreatment (>40 min) of wheat gluten led to the production of bitter and burnt off-flavors of HMPEs. Through integrated VIP ≥ 1, ROAV ≥ 1 and correlation analyses, six key odour compounds were identified: hexanal and nonanal as negative contributors, 2-pentylfuran, maltol, 2,5-dimethylpyrazine, and 2,3-diethyl-5-methylpyrazine as positive contributors. And, the excessive accumulation of 2,3-diethyl-5-methylpyrazine might be fundamentally linked to the development of burnt off flavour.

In summary, the novelty of this work lies in employing enzymatic hydrolysis of wheat gluten as an endogenous strategy to intrinsically regulate flavor formation in HMPEs by modulating flavour precursors supply and extrusion processing, which breaks through the limitation of conventional exogenous addition of minor flavour additives, providing theoretical insights and practical methods to enhance the flavour quality of alternative proteins. Future work should quantify taste-active peptides in enzymatic hydrolysates and HMPEs, and validate the identified key odorants via absolute quantification and recombination-omission tests to facilitate precise flavor tailoring for plant-based meat analogs. Additionally, larger-scale consumer preference testing is needed to directly link descriptive flavour attributes to market acceptance.

## Figures and Tables

**Table 1:** Free amino acid composition (mg/100 g) in HMPEs obtained with varying enzymatic pretreatment durations of wheat gluten.

Amino Acid	HMPE-0 min	HMPE-20 min	HMPE-40 min	HMPE-60 min	HMPE-80 min
Asp	11.25 ± 0.56 ^b^	14.89 ± 0.34 ^a^	13.41 ± 0.55 ^a^	13.45 ± 0.43 ^a^	10.54 ± 0.45 ^b^
Glu	3.40 ± 0.12 ^c^	14.40 ± 0.07 ^b^	17.65 ± 0.66 ^b^	20.08 ± 0.27 ^a^	20.58 ± 0.23 ^a^
Asn	14.22 ± 0.50 ^d^	31.25 ± 0.27 ^c^	35.17 ± 0.28 ^b^	38.00 ± 0.27 ^b^	40.04 ± 1.12 ^a^
∑UAA	28.87	60.54	66.23	71.53	71.16
Thr	2.01 ± 0.20 ^d^	21.27 ± 0.56 ^c^	23.66 ± 1.52 ^bc^	28.09 ± 0.04 ^a^	27.12 ± 0.37 ^ab^
Ser	3.78 ± 0.13 ^b^	4.79 ± 0.08 ^a^	3.88 ± 0.48 ^c^	5.40 ± 0.55 ^a^	6.42 ± 0.17 ^a^
Gly	3.68 ± 0.18 ^c^	11.77 ± 0.16 ^b^	13.41 ± 0.14 ^ab^	14.62 ± 0.71 ^a^	15.76 ± 0.53 ^a^
Ala	0.11 ± 0.01 ^d^	14.51 ± 0.56 ^c^	17.90 ± 0.56 ^b^	21.54 ± 0.18 ^a^	22.47 ± 0.49 ^a^
Pro	4.82 ± 1.45 ^b^	22.35 ± 1.61 ^a^	28.51 ± 4.23 ^a^	29.97 ± 0.45 ^a^	33.01 ± 0.19 ^a^
∑SAA	14.4	74.69	87.36	99.62	104.78
His	2.36 ± 0.29 ^c^	14.10 ± 0.31 ^a^	15.39 ± 0.21 ^a^	15.59 ± 0.05 ^a^	14.54 ± 0.26 ^a^
Arg	20.07 ± 2.25 ^a^	18.15 ± 0.93 ^a^	17.94 ± 2.47 ^a^	18.78 ± 1.02 ^a^	21.08 ± 1.34 ^a^
Tyr	3.71 ± 0.12 ^d^	29.74 ± 0.14 ^c^	37.07 ± 0.04 ^b^	42.90 ± 0.51 ^a^	46.95 ± 1.10 ^a^
Val	1.05 ± 0.22 ^d^	15.77 ± 0.16 ^c^	16.15 ± 0.13 ^bc^	17.49 ± 1.10 ^ab^	18.28 ± 0.42 ^a^
Met	0.70 ± 0.20 ^d^	15.37 ± 0.10 ^c^	19.68 ± 0.21 ^b^	23.02 ± 0.05 ^a^	25.63 ± 0.06 ^a^
Phe	1.89 ± 0.12 ^d^	4.40 ± 0.52 ^c^	10.70 ± 0.23 ^b^	11.57 ± 0.04 ^b^	12.18 ± 0.16 ^a^
Ile	1.58 ± 0.16 ^d^	117.99 ± 0.62 ^c^	142.7 ± 0.06 ^b^	161.21 ± 0.34 ^a^	172.2 ± 1.94 ^a^
Leu	1.90 ± 0.01 ^d^	6.97 ± 0.12 ^c^	8.52 ± 0.09 ^b^	9.45 ± 0.04 ^a^	9.27 ± 0.27 ^a^
Lys	332.76 ± 5.96 ^b^	359.72 ± 2.38 ^a^	348.2 ± 4.34 ^a^	346.55 ± 1.63 ^a^	348.64 ± 3.74 ^a^
∑BAA	366.02	582.21	616.35	646.56	668.77
∑TAA	409.27	717.4	769.9	817.67	844.69

Notes: UAA represents the total amount of fresh amino acids; SAA represents the total amount of sweet amino acids; BAA represents the total amount of bitter amino acids; TAA represents the total amount of amino acids; Different letters in the same line mean significant differences (*p* < 0.05).

**Table 2:** Relative contents (µg/kg) of volatile odour compounds in HMPEs obtained with varying enzymatic pretreatment durations of wheat gluten.

Compounds ^1^	RI ^2^	Threshold ^3^	HMPE-0 min	HMPE-20 min	HMPE-40 min	HMPE-60 min	HMPE-80 min
		μg/kg					
Aldehydes							
Hexanal	780	5	605.64 ± 6.50 ^a^	522.77 ± 4.30 ^b^	478.12 ± 6.39 ^b^	447.92 ± 6.23 ^c^	430.94 ± 1.88 ^c^
Heptanal	902	2.8	89.88 ± 0.48 ^a^	72.36 ± 0.99 ^b^	63.60 ± 1.01 ^c^	59.42 ± 0.77 ^c^	54.80 ± 0.95 ^c^
Benzaldehyde	960	750	199.14 ± 7.11 ^d^	441.27 ± 4.80 ^c^	489.46 ± 7.99 ^bc^	543.54 ± 4.20 ^b^	631.20 ± 4.64 ^a^
Benzeneacetaldehyde	1044	6.3	-	42.39 ± 1.08 ^c^	53.84 ± 1.58 ^bc^	61.42 ± 0.63 ^b^	74.52 ± 2.30 ^a^
Nonanal	1102	1.1	74.37 ± 0.11 ^a^	72.66 ± 1.46 ^a^	68.14 ± 2.85 ^a^	66.74 ± 0.77 ^ab^	62.89 ± 0.38 ^b^
Decanal	1205	3	29.42 ± 1.08 ^a^	25.28 ± 1.59 ^b^	25.10 ± 1.70 ^b^	28.06 ± 3.35 ^a^	26.86 ± 0.88 ^b^
Ketones							
2-Methyl-3-Octanone	985	21	24.05 ± 1.07 ^a^	-	-	-	-
2-Nonanone	1093	41	46.47 ± 0.90 ^a^	45.03 ± 2.03 ^a^	45.19 ± 0.74 ^a^	43.54 ± 1.05 ^a^	32.99 ± 1.18 ^b^
2-Decanone	1192	8.3	34.29 ± 4.77 ^a^	25.80 ± 1.66 ^b^	27.09 ± 2.25 ^b^	27.35 ± 0.85 ^b^	26.85 ± 1.97 ^b^
Alcohols							
3-Methyl-butanol	730	460	14.41 ± 0.20 ^d^	187.82 ± 3.35 ^c^	212.12 ± 2.99 ^bc^	223.05 ± 3.55 ^b^	240.15 ± 3.76 ^a^
1-Hexanol	868	5.6	20.04 ± 0.66 ^b^	35.38 ± 1.58 ^a^	23.58 ± 1.90 ^b^	26.14 ± 1.29 ^b^	24.44 ± 1.76 ^b^
1-Octen-3-ol	978	1.5	50.23 ± 0.72 ^a^	47.23 ± 0.27 ^a^	42.48 ± 1.25 ^b^	40.14 ± 0.56 ^b^	37.78 ± 0.48 ^b^
3,5-Octadien-2-ol	1037	-	29.44 ± 0.63 ^a^	29.38 ± 2.12 ^a^	26.55 ± 1.12 ^a^	25.57 ± 2.28 ^a^	24.47 ± 2.28 ^a^
Furans							
2-Ethylfuran	691	8000	168.53 ± 4.81 ^c^	188.98 ± 6.88 ^bc^	199.82 ± 0.92 ^b^	211.12 ± 7.33 ^ab^	221.90 ± 1.58 ^a^
Furfural	836	9.56	-	13.15 ± 0.36 ^c^	19.23 ± 0.83 ^c^	34.95 ± 1.57 ^b^	41.11 ± 1.09 ^a^
2-Furanmethanol	852	1900	231.90 ± 3.41 ^e^	855.95 ± 6.43 ^d^	1066.98 ± 44.52 ^c^	1298.75 ± 13.83 ^b^	1605.62 ± 27.61 ^a^
2-Pentylfuran	994	5.8	5625.80 ± 63.75 ^c^	5954.76 ± 23.9 ^bc^	6111.08 ± 23.21 ^b^	6262.06 ± 16.03 ^a^	6384.57 ± 69.71 ^a^
Maltol	1118	1.24	221.51 ± 5.74 ^d^	353.24 ± 7.12 ^c^	415.42 ± 8.46 ^b^	472.85 ± 8.06 ^a^	508.70 ± 6.93 ^a^
3-Hydroxy-2,3-dihydromaltol	1134	-	15.15 ± 0.27 ^d^	62.76 ± 1.77 ^c^	71.75 ± 1.45 ^b^	80.19 ± 1.38 ^a^	91.20 ± 2.26 ^a^
3-Phenylfuran	1228	-	-	18.61 ± 1.01 ^c^	23.19 ± 0.66 ^b c^	32.55 ± 1.81 ^b^	39.62 ± 1.16 ^a^
Thiophenes							
2-Butylthiophene	1070	-	38.87 ± 0.80 ^b^	38.62 ± 1.47 ^b^	47.73 ± 1.10 ^a^	50.65 ± 2.29 ^b^	55.06 ± 1.90 ^a^
2-Pentylthiophene	1164	-	134.60 ± 2.84 ^d^	144.47 ± 2.18 ^c^	161.48 ± 0.74 ^b^	168.95 ± 1.88 ^b^	182.80 ± 3.52 ^a^
2-Hexylthiophene	1274	-	30.65 ± 0.75 ^d^	42.66 ± 2.16 ^c^	50.95 ± 1.13 ^b^	58.20 ± 1.95 ^a^	66.17 ± 1.16 ^a^
Pyrazines							
Pyrazine	737	2	78.57 ± 0.54 ^c^	102.82 ± 6.41 ^b^	116.00 ± 0.67 ^a^	125.85 ± 3.03 ^a^	132.71 ± 1.08 ^a^
2,5-Dimethylpyrazine	916	1.75	134.30 ± 4.36 ^e^	376.42 ± 10.04 ^d^	477.71 ± 2.54 ^c^	536.83 ± 5.25 ^b^	615.53 ± 2.92 ^a^
2-Ethyl-3-methylpyrazine	1005	500	60.48 ± 0.49 ^e^	144.04 ± 8.52 ^d^	180.91 ± 2.18 ^c^	208.29 ± 5.83 ^a^	249.62 ± 6.77 ^a^
2-Ethenyl-6-methylpyrazine	1017	40	11.41 ± 1.72 ^d^	45.02 ± 2.08 ^c^	59.21 ± 1.95 ^b^	73.87 ± 2.13 ^a^	86.78 ± 1.45 ^a^
2-Acetylpyrazine	1022	60	17.08 ± 0.60 ^a^	-	-	-	-
3-Ethyl-2,5-dimethylpyrazine	1081	8.6	132.58 ± 3.33 ^d^	174.50 ± 2.29 ^c^	191.96 ± 3.23 ^b^	218.04 ± 1.42 ^a^	233.47 ± 5.60 ^a^
2,3-Diethyl-5-methylpyrazine	1200	0.0031	18.86 ± 0.12 ^d^	21.87 ± 0.65 ^c^	32.02 ± 0.23 ^b^	37.23 ± 0.77 ^a^	42.18 ± 1.04 ^a^
n-Pentylpyrazine	1216	1	8.17 ± 0.88 ^a^	-	-	-	-
2,5-Dimethyl-3-(3-methylbutyl)-Pyrazine	1308	600	34.60 ± 0.72 ^e^	198.75 ± 1.47 ^d^	254.67 ± 11.24 ^c^	295.12 ± 7.15 ^b^	322.96 ± 8.69 ^a^

^1^ Volatile odour compounds were identified based on retention index (RI) and mess information in NIST 20 database; ^2^ RI, retention index was calculated on HP-5 column using N-ketones (C6–C22); ^3^ Odour thresholds were obtained by reviewing the literature of Sohail et al. [32]. Different letters in the same line mean significant differences (*p* < 0.05).

**Table 3:** Relative odour activity value (ROAV) of volatile odour compounds in HMPEs obtained with varying enzymatic pretreatment durations of wheat gluten.

Compounds	Threshold ug/Kg	HMPE-0 min	HMPE-20 min	HMPE-40 min	HMPE-60 min	HMPE-80 min	Odour Describes
Hexanal	5	1.99	1.48	0.93	0.75	0.63	grass, green
Heptanal	2.8	0.53	0.37	0.22	0.18	0.14	fat, citrus, rancid
Benzaldehyde	750	<0.01	<0.01	<0.01	<0.01	<0.01	almond, burnt sugar
Benzeneacetaldehyde	6.3	<0.01	<0.01	<0.01	<0.01	<0.01	rosy
Nonanal	1.1	1.04	0.86	0.60	0.55	0.50	fat, citrus, green
Decanal	3	0.11	0.12	<0.01	<0.01	<0.01	fatty, rancid, burnt
2-Methyl-3-Octanone	21	<0.01	-	-	-	-	fruity, nutty
2-Nonanone	41	<0.01	<0.01	<0.01	<0.01	<0.01	flower petal, floral
2-Decanone	8.3	<0.01	<0.01	<0.01	<0.01	<0.01	heavy, sweet
3-Methyl-butanol	460	<0.01	<0.01	<0.01	<0.01	<0.01	alcohol, fruity
1-Hexanol	5.6	<0.01	<0.01	<0.01	<0.01	<0.01	minty, flower, green
1-Octen-3-ol	1.5	0.55	0.45	0.27	0.22	0.19	mushroom
2-Ethylfuran	8000	<0.01	<0.01	<0.01	<0.01	<0.01	fruity, floral
Furfural	9.56	<0.01	<0.01	<0.01	<0.01	<0.01	almond, woody
2-Furanmethanol	1900	<0.01	<0.01	<0.01	<0.01	<0.01	caramel
2-Pentylfuran	5.8	15.94	14.55	10.20	8.99	8.09	butter, caramel
Maltol	1.24	2.94	4.04	3.24	3.18	3.02	butter, caramel
Pyrazine	2	0.65	0.73	0.56	0.52	0.49	nutty
2,5-Dimethylpyrazine	1.75	1.26	3.05	2.64	2.55	2.59	popcorn, roasted
2-Ethyl-3-methylpyrazine	500	<0.01	<0.01	<0.01	<0.01	<0.01	roasted
2-Ethenyl-6-methyl- pyrazine	40	<0.01	<0.01	<0.01	<0.01	<0.01	roasted, earthy
2-Acetylpyrazine	60	<0.01	<0.01	<0.01	<0.01	<0.01	roasted
3-Ethyl-2,5-dimethylpyrazine	8.6	0.25	0.29	0.22	0.21	0.20	nutty, roasted
2,3-Diethyl-5-methylpyrazine *	0.0031	100	100	100	100	100	roasted, burnt
n-Pentylpyrazine	1	<0.01	<0.01	<0.01	<0.01	<0.01	roasted
2,5-Dimethyl-3-(3-methylbutyl)-pyrazine	600	<0.01	<0.01	<0.01	<0.01	<0.01	roasted

* 2,3-Diethyl-5-methylpyrazine was employed as the normalized reference substance to calculate the relative odour activity value (ROAV) for volatile odour compounds in HMPEs.

## Data Availability

The original contributions presented in this study are included in the article/Supplementary Material. Further inquiries can be directed to the corresponding authors.

## Supplementary Materials

The following supporting information can be downloaded at: https://www.mdpi.com/article/10.3390/foods15050912/s1, Table S1: Criteria for sensory evaluation.
