# Supplementary material for: The Impact of Varying Enzymatic Pretreatment Durations of Wheat Gluten on the Flavour Characteristics of High-Moisture Plant-Based Extrudates
Source: Foods. 2026 Mar 6;15(5):912. doi: 10.3390/foods15050912 (PMC12984281; doi:10.3390/foods15050912)
Supplement: Supplementary file 1 [file foods-15-00912-s001.zip › foods-4133267-supplementary.pdf]

Table S1 Criteria for sensory evaluation

Project	Standard description	Score
Fibrous structure	Abundant, well-oriented fibrous structure, forms long, continuous strands upon tearing.	9~10
	Clearly visible fibrous structure, but may be short, discontinuous, or slightly too dense.	6~8
	Weak fibrous structure, loose with some pores, breaks easily upon tearing.	3~5
	Essentially no fibrous structure, appears as a dense sheet or loose granules.	0~2
Texture	Optimal hardness and elasticity, good chewiness, juicy and pleasant mouthfeel.	9~10
	Slightly insufficient or excessive hardness/elasticity, acceptable chewiness, somewhat juicy.	6~8
	Obviously too soft or hard, lacks elasticity, poor chewiness, not noticeably juicy.	3~5
	Extremely soft/hard, no elasticity, difficult to chew, dry and unpleasant.	0~2
Color	Uniform color, attractive light/dark brown, bright and shiny surface.	9~10
	Uniform color, but slightly dull gloss or slightly dark color.	6~8
	Non-uniform color, or obvious dark color.	3~5
	Abnormal color (e.g., overly dark blackened).	0~2
Taste	Intense, harmonious umami and sweetness, free of bitterness or other off-tastes.	9~10
	Distinct umami and sweetness, without any unpleasant taste.	6~8
	Weak umami and sweetness, or slight bitterness begins to appear.	3~5
	Basically no umami/sweetness, obvious bitterness or other unpleasant tastes.	0~2
Odor	Intense, pleasant roasted/ beany aroma, free of off-odors.	9~10
	Distinct roasted or beany aroma, slight burnt odor.	6~8
	Weak aroma, obvious beany off-flavor or burnt odor.	3~5
	No pleasant aroma, strong beany, burnt or other off-flavor.	0~2
